# The effectiveness and safety of acupuncture in the treatment of lumbar disc herniation: Protocol for a systematic review and meta-analysis

**DOI:** 10.1097/MD.0000000000018930

**Published:** 2020-03-20

**Authors:** Rong Deng, ZiLing Huang, Xun Li, XingHong Pei, ChengXi Li, JianXin Zhao

**Affiliations:** a The Third Affiliated Hospital of Beijing University of Traditional Chinese Medicine; b Centre for Evidence-Based Chinese Medicine, Beijing University of Chinese Medicine, Beijing, China.

**Keywords:** acupuncture, lumbar disc herniation, protocol, systematic review

## Abstract

**Background:**

Lumbar disc herniation (LDH) is a common disease that seriously affects patients’ quality of life. Although several articles have reported that acupuncture can improve the symptoms of LDH, different guidelines do not evaluate the efficacy of acupuncture consistently, new randomized controlled trials have been published in recent years.

The purpose of this study is to evaluate the efficacy and safety of acupuncture for LDH.

**Method:**

Electronic resource databases, trial registration platform, and different types of grey literature will be systematically searched for eligible studies by 2 authors independently. The type of trial will be limited to randomized controlled trials on acupuncture treatment for LDH. Search strategy will be a combination of terms associated with LDH (eg, low back pain or sciatica) and study of design (eg, randomized controlled trials or clinical trial). Data from homogeneous studies will be combined in a fixed-effects model, and the evidence level will be measured by grading of recommendations assessment, development, and evaluation.

**Results:**

This study will provide high-quality evidence to evaluate the relief of pain intensity and improvement of dysfunction of acupuncture in patients with LDH, and to evaluate the safety of acupuncture.

**Conclusion:**

This study will provide strong evidence for evaluating whether acupuncture therapy is effective and safe for LDH patients.

**PROSPERO registration number:**

CRD 42019137399.

## Abstract 2

Supplemental Digital Content is available in the text

## 1 Introduction

Lumbar disc herniation (LDH) is a common degenerative spinal disease that affects patients’ quality of life and ability to work. According to the World Health Organization (WHO), LDH has become a major cause of disability in both developed and developing countries.^[1]^ The main symptoms of LDH are low back pain (LBP) and sciatica. Two-thirds of adults have suffered back pain in their lives,^[2]^ and approximately 85% of patients suffered sciatica caused by LDH.^[3]^ LDH has put a heavy burden on individuals, families, and society.^[4]^

The treatment of LDH includes surgical and non-surgical treatment, and patients suitable for surgery account for only 10% to 20% of all patients.^[5]^ Although surgery can quickly relieve the pain intensity, in the long run, there was no significant difference in functional improvement and pain relief between surgery and conservative treatment^[6]^; 5 years after surgery, 7% of patients relapsed or had recurrent surgical indications.^[7]^ The operation may cause nerve root adhesion,^[8]^ dorsal root ganglion injury, dural membrane tears, and other side effects. For patients without surgical indications, conservative therapy should be selected, as it provides good efficacy, has minor side effects and is more economical.^[3]^ Acupuncture, as an effective, safe and economical treatment, is widely used in clinical practice. In 2002, the WHO recommended 107 indications for acupuncture, including LBP and sciatica caused by a herniated disc.^[9]^ A large number of articles have reported that acupuncture can relieve pain intensity and improve function in patients with LDH.^[10–12]^ However, there is a lack of high-quality evaluations of the efficacy and safety of acupuncture, different guidelines do not evaluate the efficacy of acupuncture consistently, new randomized controlled trials have been published in recent years. Thus, this study will evaluate the effectiveness and safety of acupuncture for treating LDH.

## 2 Methods

### 2.1 Design and registration of the review

This systematic review and meta-analysis protocol has been registered at PROSPERO. The registration number is CRD 42019137399. This systematic review protocol is structured in accordance with the preferred reporting items for systematic reviews and meta-analysis protocols statement guidelines.^[13]^

### 2.2 Inclusion criteria for study selection

#### 2.2.1 Type of study

Randomized controlled trials (RCTs) are the only study type to be included. Quasi-RCTs, review articles, case reports, and other studies that do not meet the requirements will be excluded.

#### 2.2.2 Types of participants

Patients diagnosed with LDH by magnetic resonance imaging or computed tomography will be included. Patients with other diseases that cause pain in the lower back or legs will be excluded, such as spinal tumors, cauda equina syndrome, recent fractures/joint dislocations, spondylolisthesis, spinal stenosis, spinal infections, abdominal aneurisms, cancer, unexplained weight loss, severe or progressive neurological deficits, fibromyalgia, and rheumatoid arthritis. Patients who are pregnant will also be excluded.

#### 2.2.3 Types of interventions

Interventions involving the insertion of needles into the skin, but not for the purpose of injection, will be included, for example, acupuncture, electroacupuncture, and abdominal acupuncture will be included, but hydro-acupuncture will be excluded. Acupuncture that does not involve needle insertion (such as laser acupuncture) will also be excluded. Interventions combining acupuncture with other treatments will be included, but interventions combined with traditional Chinese medicine or other types of acupuncture will not. Moreover, interventions compared between different types of acupuncture will be excluded. The treatment frequency, treatment method, and course of treatment are not limited.

Comparison groups will include rehabilitation therapy, kinetotherapy, manipulative therapy, physical therapy, drug therapy (eg, nonsteroidal anti-inflammatory drugs), and surgery (eg, discectomy, interbody fusion). Blank controls and sham controls will also be included. Trials using traditional Chinese medicine as a control will be excluded.

#### 2.2.4 Types of outcome measures

Pain intensity will be measured as the main outcome. There are no restrictions on the scale used to measure pain intensity.

Functional status, quality of life, depression status, and anxiety status will be measured as well, any adverse events in the included studies will be assessed.

### 2.3 Data sources

Electronic resource databases, trial registries, retrospective references and different types of grey literature will be the main sources of information.

Electronic resource databases including Web of Science, Cochrane Library, PubMed, Excerpta Medica Database, Wanfang Database, technology journal, China National Knowledge Infrastructure, Chinese Biomedical Literature database will be searched for potentially eligible studies. Other types of articles (guidelines, reviews, meta-analyses, and academic dissertations etc) will be searched, and guidelines will also be searched in the National Guideline Clearinghouse. ClinicalTrials.gov and the WHO International Clinical Trials Registry Platform will be searched for on-going registered trials. Grey literature will be searched in OpenGrey.

The search will begin in August 2019, and for each database, the publication period will be set from their inception until August 2019. The language will be limited to Chinese or English, and the search strategy will be provided as an attachment.

### 2.4 Search strategy

The search strategy is created on the basis of the Cochrane handbook guidelines (5.1.0). The search keywords or combination subject terms will include the following: herniated disc, herniated disk, disc herniation, disk herniation, slipped disc, slipped disk, intervertebral disc displacement, intervertebral disk displacement, and RCTs. The corresponding search terms will be used in the Chinese databases. The strategy for searching the PubMed database is attached in Supplemental Digital Content (Appendix 1).

### 2.5 Data collection and analysis

#### 2.5.1 Selection of studies

**Figure 1:**
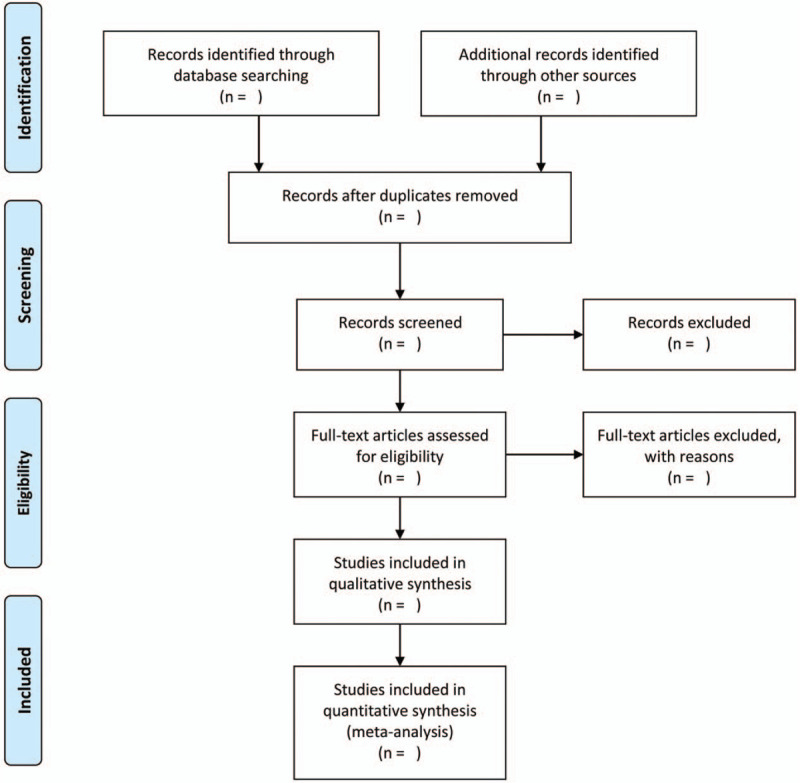
The PRISMA flow chart of the selection process. PRISMA = preferred reporting items for systematic review and meta-analysis.

The retrieved literature will be imported into the NoteExpress (V 3.0.4.6732) library and duplicate articles will be eliminated. Two reviewers (RD and ZLH) will independently scan the titles and abstracts to omit articles do not meet the inclusion criteria. Then, the same 2 reviewers will independently read the full text of the articles to assess their eligibility. Any discrepancies will be settled by discussion and as needed, a third independent reviewer (JXZ) will serve as an arbitrator and ultimately make the decision. The selection process is shown in the preferred reporting items for systematic reviews and meta-analysis flow chart in Figure 1.

#### 2.5.2 Data extraction and management

Data from the selected articles will be independently entered into an Excel spreadsheet by 2 reviewers (XHP and CXL). The extracted information will include the reference ID, name of the lead author, publication period, country, participant characteristics, intervention, sample size, blinding method, randomization method, outcome measures, duration of follow-up, adverse effects, and other detailed information. Other necessary information will be complemented by contacting the author.

#### 2.5.3 Risk of bias assessment and study quality

The Cochrane Collaboration's risk-of-bias tool will be used for evaluating the risk of bias, which will be independently evaluated by 2 reviewers. The risk of bias will be assessed in 6 dimensions: random sequence generation; allocation concealment; blinding method for patients, researchers and outcome evaluators; incomplete result data; selective reporting; and other issues. The degree of the risk of bias will be classified into 3 levels: low risk, unclear risk, and high risk. Any discrepancies will be resolved through discussions with the third author. When a consensus cannot be reached by discussion, the third reviewer will make the decision.

#### 2.5.4 Measurement of the treatment effect

Methods vary depending on the type of data. For continuous data, the mean difference and 95% confidence interval (CI) will be used.^[14]^ For numerical data, the relative risk and absolute risk reduction with 95% CI will be used. Adverse events will be described by tables.

#### 2.5.5 Management of missing data

Missing data will be supplemented by contacting the author; the waiting time defaults to 1 month after an email is sent.

#### 2.5.6 Assessment of heterogeneity

According to the Cochrane Handbook for Systematic Reviews of Interventions,^[15]^*I*^2^ statistics will be used to evaluate heterogeneity.^[16]^ If *I*^2^ < 50%, the heterogeneity will be considered to be minor, and a fixed-effects model will be used. If *I*^2^ > 50%, the heterogeneity will be considered to be significant, Subgroup analysis or sensitivity will be performed to explore the reasons underlying the heterogeneity, and random effect models will be used for data analysis.

#### 2.5.7 Assessment of reporting biases

If more than 10 trials were included in study, the visual asymmetry on the funnel plot will be used to evaluate the reporting bias. If funnel plot asymmetry is detected, the reasons for this outcome will be analyzed.

#### 2.5.8 Data synthesis

Review Manager (Version 5.3. Copenhagen: The Nordic Cochrane Centre, The Cochrane Collaboration, 2014) will be used for statistical synthesis and analyses. A fixed-effects model or random-effects model will be used based on the heterogeneity levels of the included studies. Fixed effects models are used for data with no statistical heterogeneity, and random-effects models with 95% CIs will be used to analyze the pooled effect for data with statistical heterogeneity. When there is significant heterogeneity, subgroup analysis or sensitivity analysis will be use to find the source of heterogeneity. If the source of heterogeneity is unknown, only descriptive analysis will be performed.

#### 2.5.9 Subgroup analysis

If significant heterogeneity (*I*^2^ > 50%) exists, and more than 10 trials were included, subgroup analysis will be performed to identify the sources of heterogeneity.

#### 2.5.10 Sensitivity analysis

A funnel plot will be used in the sensitivity analysis to assess the reliability of this review, and the evaluation dimensions will include the sample size, heterogeneity qualities, and type of statistical model (random-effects model or fixed-effects model).

#### 2.5.11 Grading the quality of evidence

The quality of evidence will be measured by grading of recommendations assessment, development and evaluation and classified into 4 levels: very low, low, moderate, and high.^[17]^

#### 2.5.12 Ethics and dissemination

This is a literature-based study, ethical approval is not requires for this protocol.

## 3 Discussion

LDH is a common cause of LBP and activity limitations in young and middle-aged individuals.^[18]^ Acupuncture is widely used for LDH, and its efficacy has been recognized by the WHO since 2002. However, in the past 5 years, doubts surrounding the efficacy of acupuncture still exist.^[19]^

According to the theory of traditional Chinese medicine, pain is caused by obstruction of Qi and the lack of nutrition. Acupuncture can quickly unclog the meridians and Qi, thus achieving the effect of “the general principles of the pain.” According to modern medical research, physical stress, chemical stimulation from an inflammatory response, microcirculation disturbance, or nerve root edema to the extent of nucleus pulpotomy are the causes of LDH leading to LBP and sciatica.^[18]^ By stimulating the nerve trunk, acupuncture can relieve the high-tension state of the nerve and the structural relationship between the nerve and the lumbar disc to relieve the symptoms of sciatica.

The incidence of disc herniation increases with age,^[20]^ and the incidence of LDH has been increasing, showing a trend of younger age.^[21]^ Acupuncture, which is widely used in clinical practice with fewer side effects, is a promising treatment. This systematic review and meta-analysis will provide patients, clinicians, and health decision makers with a deeper understanding of the efficacy and safety of acupuncture.

## Author contributions

RongDeng is the guarantor of the article. RD and ZLH drafted and revised the article; XL provided assistance in the formation of retrieval strategies, XHP and CXL will extract data independently, RD and ZLH will assess the risk of bias and conduct data synthesis. JianXin Zhao supervize and guide all the work for this paper. All authors approve the publication of the protocol.

## Correction

In the original publication, the funding information for this research appeared incorrectly as ‘the National Natural Science Foundation of China [grant number 81373731]’ and has been corrected to Beijing Municipal Science and Technology Commission , Award ID: Z161100000516137’.

## Supplementary Material

Supplemental Digital Content
