# Supplementary material for: The effectiveness and safety of acupuncture in the treatment of lumbar disc herniation: Protocol for a systematic review and meta-analysis
Source: Medicine (Baltimore). 2020 Mar 20;99(12):e18930. doi: 10.1097/MD.0000000000018930 (PMC7220427; doi:10.1097/MD.0000000000018930)
Supplement: Supplemental Digital Content [file medi-99-e18930-s001.doc]

The strategy for searching the PubMed database

#1 herniated disk[Title/Abstract]

#2 herniated disk[MeSH Terms]

#3 herniated disc[Title/Abstract]

#4 herniated disc[MeSH Terms]

#5 slipped disc[Title/Abstract]

#6 slipped disc[MeSH Terms]

#7 slipped disk[Title/Abstract]

#8 slipped disk[MeSH Terms]

#9 disc herniation[Title/Abstract]

#10 disc herniation[MeSH Terms]

#11 disk herniation[Title/Abstract]

#12 disk herniation[MeSH Terms]

#13 intervertebral disc displacement[Title/Abstract]

#14 intervertebral disc displacement[MeSH Terms]

#15 intervertebral disk displacement[Title/Abstract]

#16 intervertebral disk displacement[MeSH Terms]

#17 lumbar[Title/Abstract]

#18 lumbar[MeSH Terms]

#19 ((#1 OR #2 OR #3 OR #4 OR #5 OR #6 OR #7 OR #8 OR #9 OR #10 OR #11 OR #12 OR #13 OR #14 OR #15 OR #16) AND (#17 OR #18))

#20 backache[Title/Abstract]

#21 backache[MeSH Terms]

#22 back pain[Title/Abstract]

#23 back pain[MeSH Terms]

#24 dorsalgia[Title/Abstract]

#25 dorsalgia[MeSH Terms]

#26 lumbago[Title/Abstract]

#27 lumbago[MeSH Terms]

#28(#20 OR #21 OR #22 OR #23 OR #24 OR #25 OR #26 OR #27)

#29 sciatica[Title/Abstract]

#30 sciatica[MeSH Terms]

#31 ischialgia[Title/Abstract]

#32 ischialgia[MeSH Terms]

#33 radicular pain[Title/Abstract]

#34 radicular pain[MeSH Terms]

#35 scitic[Title/Abstract]

#36 scitic[MeSH Terms]

#37(#29 OR #30 OR #31 OR #32 OR #33 OR #34 OR #35 OR #36)

#38(#19 OR #28 OR #37)

#39 acupuncture[Title/Abstract]

#40 acupuncture[MeSH Terms]

#41 neddle[Title/Abstract]

#42 neddle[MeSH Terms]

#43( #39 OR #40 OR #41 OR #42)

#44 (randomized controlled trial[pt] OR controlled clinical trial[pt] OR randomized[tiab] OR placebo[tiab] OR clinical trials as topic[mesh:noexp] OR randomly[tiab] OR trial[ti]) NOT (animals[mh] NOT humans[mh])

#45(#38 AND #43 AND #44)
